# Functional analysis of human T lymphotropic virus type 2 Tax proteins

**DOI:** 10.1186/1742-4690-3-20

**Published:** 2006-03-21

**Authors:** Noreen Sheehy, Lorraine Lillis, Karen Watters, Martha Lewis, Virginie Gautier, William Hall

**Affiliations:** 1 Centre for Research in Infectious Disease, School of Medicine & Medical Science, University College Dublin, Belfield, Dublin 4, Ireland; 2 University of California, Department of Medicine, UCLA Centre for Health Sciences, Los Angeles, California, USA

## Abstract

**Background:**

The Tax proteins encoded by human T lymphotropic virus type 1 (HTLV-1) and type 2 (HTLV-2) are transcriptional activators of both the viral long terminal repeat (LTR) and cellular promoters via the CREB and NFkB pathways. In contrast to HTLV-1, HTLV-2 has been classified into four distinct genetic subtypes A, B, C and D defined by phylogenetic analysis of their nucleotide sequences and the size and amino acid sequence of their Tax proteins. In the present study we have analysed and compared the transactivating activities of three Tax 2A and one Tax 2B proteins using LTR and NFkB reporter assays.

**Results:**

We found that with the exception of the prototype Tax 2A Mo protein, the other two Tax 2A proteins failed to transactivate either the viral LTR or NFkB promoter in Jurkat and 293T cells. Loss of activity was not associated with either expression levels or an alteration in subcellular distribution as all Tax 2 proteins were predominantly located in the cytoplasm of transfected cells. Analysis of the sequence of the two inactive Tax 2A proteins relative to Mo indicated that one had six amino acid changes and the other had one change in the central region of the protein. Mutations present at the amino and the extreme carboxy termini of Mo resulted in the loss of LTR but not NFkB activation whereas those occurring in the central region of the protein appeared to abolish transactivation of both promoters. Analysis of the transactivation phenotypes of Tax 1, Tax 2A Mo and Tax 2B containing mutations identified in the present study or previously characterised Tax mutations showed that domains required for LTR and NFkB activation are very similar but not identical in all three Tax proteins.

**Conclusion:**

Our results suggest that loss of activity of two Tax 2A proteins derived from different isolates is associated with multiple amino acid changes relative to Mo in domains required for the activation of the CREB or CREB and NFkB pathways and that these domains are very similar but not identical in Tax 2B and Tax 1. The loss of Tax function in 2A viruses may have implications for their biological and pathogenic properties.

## Background

HTLV-1 and HTLV-2 are closely related human retroviruses which have a preferential *in vivo *tropism for CD4 + and CD8 + T lymphocytes respectively. HTLV-1 is the causative agent of adult T cell leukaemia (ATL) and a neurodegenerative disorder, tropical spastic paraparesis or HTLV-1 associated myelopathy (TSP/HAM) [1-4]. In contrast, the role of HTLV-2 in human disease is less clearly defined; however increasing evidence suggests that infection may also be associated with rare lympho-proliferative and neurological disorders [5-7].

In addition to the essential retroviral proteins Gag, Pol and Env, HTLV encodes a number of regulatory and accessory proteins that modulate viral gene expression and play important roles in viral pathogenesis. The most widely studied of these is the transactivating protein Tax [8]. Tax is known to alter cellular signalling pathways by interacting with a number of cellular transcription factors including activating transcription factor/c-AMP response element-binding protein (ATF/CREB) and NFkB. Specifically Tax enhances transcription of the viral genome by interacting with CREB/ATF which increases its affinity for conserved binding sites within the LTR and cellular promoters. With respect to the NFkB pathway, cytoplasmic Tax acts by binding the IKK γ which induces the phosphorylation and degradation of IkB-α, the inhibitor of NFkB, thereby allowing the NFkB complex to migrate to the nucleus and induce gene expression.

The different subtypes of HTLV-1 encode Tax proteins (Tax 1) of equal lengths. In contrast, HTLV-2 has four distinct genetic subtypes, A, B, C and D, defined by phylogenetic analysis of their nucleotide sequences and the size and amino acid sequence of their Tax proteins. The Tax proteins of HTLV-2 (Tax 2) vary in length, with Tax 2B and -2C having similar lengths to Tax 1, 356 and 353 amino acids respectively, although the C-terminal sequences of these proteins are divergent [9,10]. Tax 2A lacks a 25 amino acid C terminal sequence having a stop codon which truncates the protein at amino acid 331. HTLV-2D encodes a Tax protein of 344 amino acids that as yet remains uncharacterised [11]. Studies comparing the relative transactivation functions of Tax 1 and Tax 2 indicate that, with the exception of Tax 2A, there are no significant differences in transactivation activities via CREB and NFκB pathways between the Tax proteins of these two viruses and suggest that Tax 2B may have the same pathogenic potential as Tax 1 [12].

Several studies have identified functional domains in Tax 1 which are required for NFkB and LTR activation. These regions include activation domains at the amino and carboxy termini, a CREB binding domain and zinc binding domain within the first 60 amino acids [13,14]. Tax 1 and Tax 2 contain nuclear localization signals (NLS) at the amino terminus between amino acids 1–60 [15] and 1–40 [16], respectively, and nuclear export signals (NES) located between amino acids 188 and 202 [17,18]. Using mutations previously characterised in Tax 1, Tax 2A was found to contain similar but not identical functional domains as Tax 1 [19]. Various studies reported that Tax 1 shuttles between the nucleus and the cytoplasm, and depending on the cell line is predominantly located in the nucleus [20,21]. A recent study has shown that in contrast to Tax 1, Tax 2A and Tax 2B are predominantly found in the cytoplasm of either a HTLV-2 infected cell line or cells transfected with Tax 2 expression plasmids [22]. Using chimeric plasmids containing domains from Tax 1 and Tax 2 it could be shown that amino acids 90 to 100 are involved in the cytoplasmic localization of Tax 2.

In a previous study we reported that some Tax 2A proteins exhibit poor transactivation of both the CREB and NFkB pathways and this appeared to be related to decreased levels of Tax 2A expression [12]. The aims of the present study were firstly, to examine the ability of different Tax 2A proteins to transactivate the viral LTR and a NFkB promoter in relation to expression levels, sequence variation and sub-cellular distribution and secondly, to determine if Tax 2A and Tax 2B have similar functional domains. We show that two Tax 2A proteins were non-functional relative to the prototype 2A Mo protein in either Jurkat or 293T cells. Loss of activity was not correlated with Tax 2A expression levels or altered sub cellular distribution but appears to be due to the presence of amino acid changes. We identified previously uncharacterised mutations in the non-functional Tax 2A proteins that result in either defective LTR and NFkB activation or defective LTR but not NFkB activation. These mutations resulted in similar but not identical transactivation phenotypes in Tax 2B.

## Results

### Transactivation phenotypes of Tax 2A Lor and Gar

In the present study we examined the transactivation phenotypes of two Tax 2A proteins Lor and Gar and compared this with the prototype 2A isolate, Mo. Lor was derived from a HTLV IIA infected cell line and Gar was derived from cultured PBMCs from a HTLV-2/HIV-1 co-infected patient (W. Hall unpublished). All Tax coding sequences were cloned in the same expression plasmid and were tagged with a HIS tag to allow the simultaneous detection of all Tax proteins. A HTLV-1 LTR-LUC reporter was used in this study to assess the activity of Tax 2 proteins as previous studies have shown that there is no significant difference in the ability of Tax 2 proteins to activate the LTR from either HTLV-1 or HTLV-2 [19]. Functional assays were performed in Jurkat cells as these cells are lymphocytes and represent the natural targets of HTLV *in vivo*.

**Figure 1:**
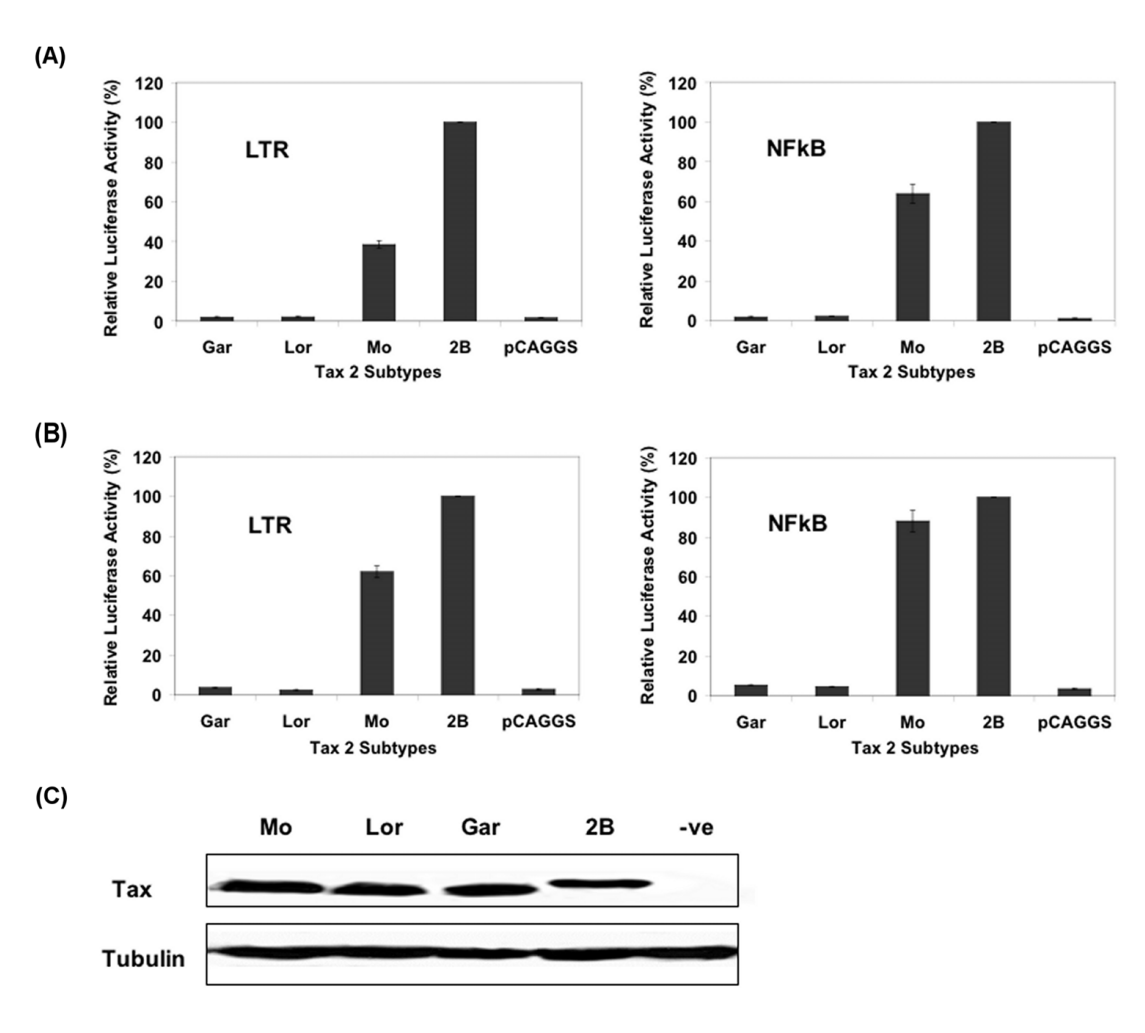
Relative transactivation phenotypes and expression levels of Tax 2A proteins Mo, Lor, Gar and Tax 2B proteins. Jurkat (A) and 293T cells (B) were co-transfected with 250 ng of empty or Tax expression plasmids together with 1ug of either the HTLV-1 LTR or NFkB luciferase reporter plasmids and 50 ng of pRL-TK. Reporter activities were measured using the Dual Luciferase Assay system (Promega) and were normalised to Renilla luciferase values. The values indicate the mean of four independent experiments normalised to Tax 2B (100%) and the error bars represent the SEM. A minimum of three replicates of each Tax construct was included in the calculation of each mean activity. (C) Western blot analysis of lysates from 293T cells transfected with 250 ng of the indicated plasmids. Tax proteins were detected using an anti-HIS antibody. Tubulin was used as a loading control and was detected using anti-Tubulin.

In initial studies we employed well characterised Tax mutants in our assays as had been reported in other studies. Specifically we tested the transactivation activities of the Tax 2A Mo mutants designated M22 (S130A/L131F), which was shown in previous studies to result in LTR but not NFkB activation, and M47 (I319R/L320S), which was shown to abolish activation of the LTR by Tax 2A Mo while not affecting NFkB activation [13,14]. These mutants displayed the expected transactivation phenotype (Table 1). Similar results were also obtained with the Tax 2B M22 and Tax 2B M47 mutants. Tax 2A Lor and Gar failed to transactivate either the viral LTR or NFkB promoters in Jurkat and 293T cells compared to the prototype Tax 2A protein Mo or Tax 2B (Figure 1A and 1B, respectively). Wildtype Mo was repeatedly found to activate the LTR and NFkB promoters less efficiently than Tax 2B, for example 60% and 40% in Jurkat cells and 40% and 20% in 293T cells, respectively. Mo, Lor, Gar and Tax 2B were all expressed at similar levels in 293T cells (Figure 1C).

**Table 1:** Transactivation phenotypes of previously characterised Tax mutants in Jurkat cells

		Mutation	LTR	NFkB
Mo	WT	None	100%	100%
		M22 (S130A/L131F)	105%	< 10%
		M47 (I319R/L320S)	< 5%	130%
2B	WT	None	100%	100%
		M22 (S130A/L131F)	110%	< 5%
		M47 (I319R/L320S)	< 5%	115%

Jurkat cells were co-transfected with 250 ng of the indicated Tax 2 plasmids, 1 ug of the LTR or NFkB luciferase reporters and 50 ng of the control Renilla plasmid pRL TK. After 24 hrs, the cells were lysed and reporter activities were measured using a Turner 20/20 Luminometer. Firefly reporter activities were normalised using Renilla luciferase values. Normalised values for mutant Tax proteins were calculated as a percentage of the wildtype Mo or Tax 2B values which was set at 100%.

### Sub cellular localisation of Tax 2 proteins

**Figure 2:**
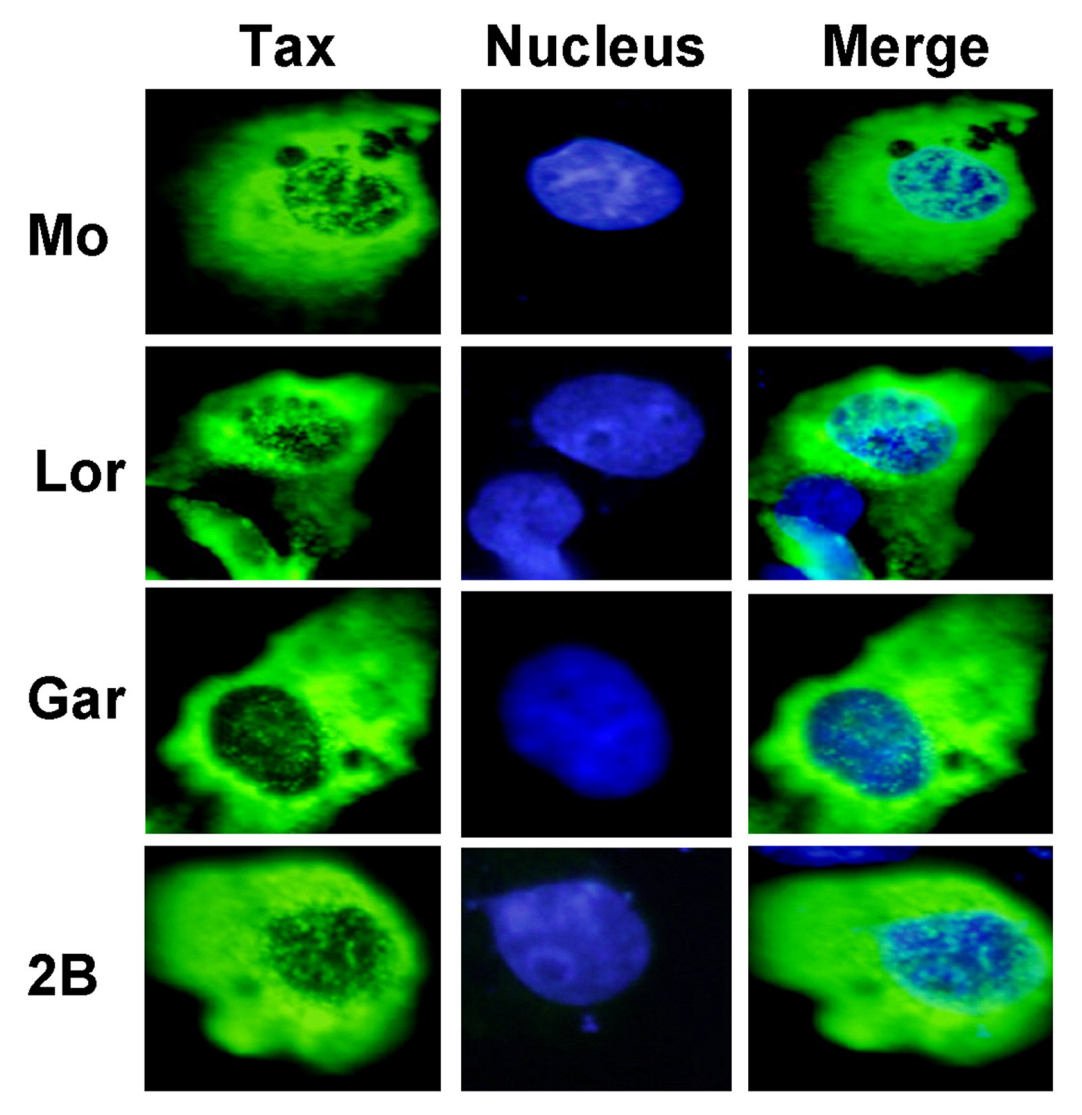
Intracellular location of Tax 2 proteins in Cos 7 cells. Immunofluorescence was performed on cells transfected with 150 ng of the indicated Tax expression plasmids. Tax expression was detected using an anti-HIS antibody followed by an anti mouse FITC secondary antibody. The cell nuclei were stained with DAPI. The green signal represents Tax expression and the blue signal corresponds to DAPI.

A previous study demonstrated that Tax function was related to its sub-cellular localisation, with the highest levels of LTR and NFkB activity being observed when Tax was predominantly located in either the nucleus or cytoplasm, respectively [22]. We sought to determine if the intracellular distribution of Lor and Gar was altered compared to that of Mo and Tax 2B. Immunofluorescence studies showed that Gar and Lor were found predominantly in the cytoplasm but also appeared as intense specks in the nucleus of 293T cells (data not shown) and Cos 7 cells and displayed a similar intra cellular distribution as Mo and Tax 2B (Figure 2). These results clearly indicate that the sub cellular distribution of Lor or Gar was not contributing to their loss of activity.

### Sequence analysis of Lor and Gar Tax proteins

**Figure 3:**
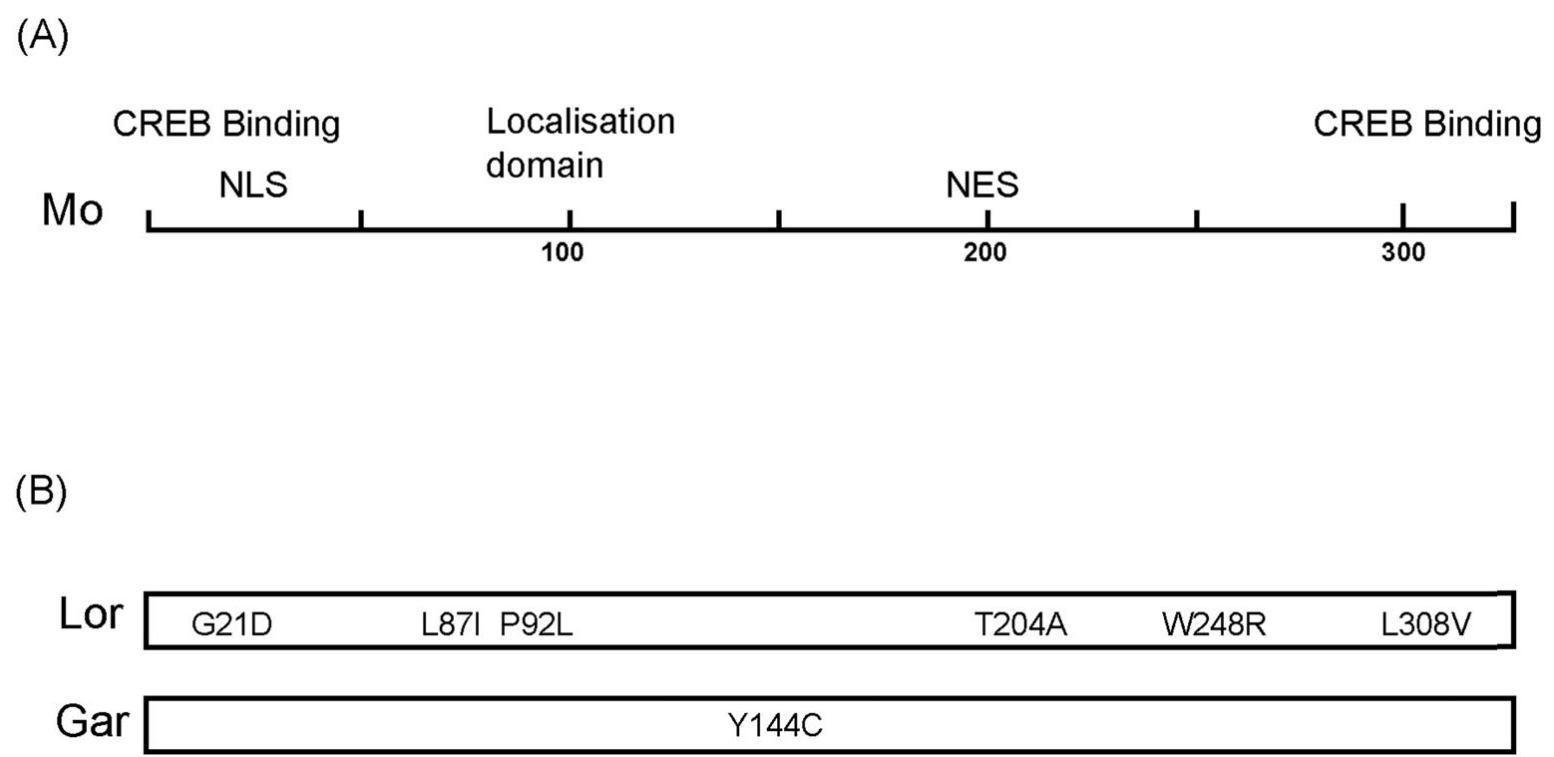
Location of mutations found in Tax 2A proteins. (A) Schematic representation of functional domains in Tax 2A Mo. These regions include CREB binding domains at the amino and carboxy termini, domains important in CREB and NFkB activation flanking a domain found to be important in NFkB activation [19], a domain involved in the cytoplasmic localisation of Tax 2 proteins [22], a nuclear localisation domain (NLS) [16] and a nuclear export signal (NES) [18]. Mutations which give rise to the loss of activation of CREB alone, NFkB alone or CREB and NFkB pathways by Mo are indicated. (b) Relative position of amino acid changes found in Lor and Gar.

The sequences of Lor and Gar were determined and compared to that of Mo. Lor had six amino acid changes spanning the entire protein at positions G21D, L87I, P92L, T204A, W248R and L308V (Figure 3B). Gar only contained one amino acid change at position Y144C. G21D and L308V are located in a domain previously found to be involved in LTR activation while L87I and P92L are close to a domain previously found to be involved in the cytoplasmic localization of Tax 2 proteins (Figure 3A) [19,22]. W248R and Y144C are located in the central region of Mo which was shown in previous studies to be important in the activation of both CREB and NFkB pathways by Mo [19].

### Ability of Tax 2A mutants to transactivate the HTLV-1 LTR and NFkB promoters

**Figure 4:**
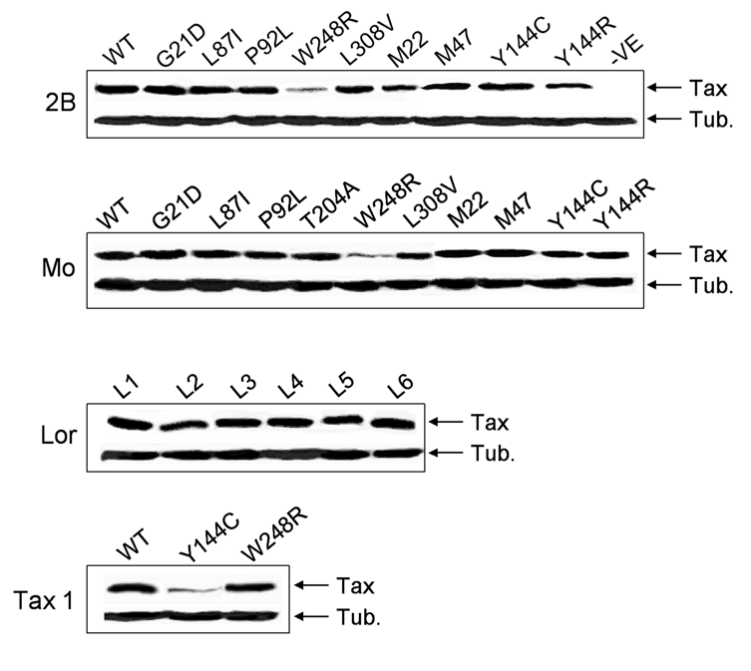
Expression levels of Tax 2 wildtype and mutant proteins. 293T cells were transfected with either wildtype or mutant Tax plasmids and cell lysates were subjected to electrophoresis on 10% SDS polyacrylamide gels. Western blots were performed using anti-HIS to detect Tax expression and anti-tubulin to detect Tubulin which was used as a loading control. Each panel shows the expression levels of both wildtype (WT) and corresponding mutant proteins for the indicated Tax proteins.

Initially site directed mutagenesis was used to sequentially replace each mutation present in Lor with the corresponding wildtype Mo residues starting from the amino terminus (Table 2; L1 to L5). Activation of both the LTR and NFkB promoters was only restored in Lor L5 when the mutation at position W248R was replaced by the corresponding wildtype Mo residue indicating that this position is critical for Tax 2A activity. Lor L6, which contains all the mutations found in Lor except for W248R, failed to activate the LTR while displaying wildtype levels of NFkB activity. All Lor mutants were expressed at similar levels (Figure 4). Insertion of individual mutations found in Lor into Mo showed that most mutations and particularly G21D, L87I, and P92L substantially reduced the ability of Mo to transactivate the LTR and without affecting NFkB activity (Table 3). Analysis of the subcellular location of mutant proteins in Cos 7 cells using immunofluorescence did not reveal any discernable alterations in their distribution relative to wildtype Mo (data not shown). The mutation L308V did not appear to affect the ability of Mo to transactivate either promoter. One mutation at position T204A appeared to enhance the ability of Mo to activate both the LTR and NFkB promoters to levels above those obtained with Tax 2B. This mutant was expressed at a similar level to wildtype Mo (Figure 4). As expected the mutation at position W248R abolished the ability of Mo to activate either the LTR or NFkB promoters. However this mutant appeared to be expressed at a lower level than wildtype Mo or other Mo mutants (Figure 4). Insertion of the only mutation found in Gar Y144C into Mo abolished its ability to activate either the LTR or NFkB promoters. To determine if the residue at position Y144, and not only the mutation Y144C, is important for Mo activity an arginine instead of a cysteine was introduced at this position. Mo Y144R displayed the same phenotype as Y144C indicating that this position is important for Mo activity irrespective of which residue is present. Insertion of Y144C into Tax 1 only reduced its activity while W248R abolished both LTR and NFkB activation by Tax 1. While Tax 1 W248R was expressed at a similar level to Tax 1 WT, Tax 1 Y144C was very poorly expressed (Figure 4).

**Table 2:** Transactivation phenotypes of Tax 2A Lor mutants

	Mutations	LTR	NFkB
Mo	None	100%	100%
Lor	G21D/L87I/P92L/T204A/W248R/L308V	< 5%	< 5%
L1	L87I/P92L/T204A/W248R/L308V	< 5%	< 5%
L2	P92L/T204A/W248R/L308V	< 5%	< 5%
L3	T204A/W248R/L308V	< 5%	< 5%
L4	W248R/L308V	< 5%	< 5%
L5	L308V	75%	80%
L6	G21D/L87I/P92L/T204A/L308V	< 5%	150%

Jurkat cells were co-transfected with either 250 ng of Mo wildtype, Lor or Lor mutants (L1–L6) together with 1 ug of the LTR or NFkB luciferase reporters and 50 ng of the control Renilla plasmid pRL TK. After 24 hrs the cells were lysed and reporter activities were measured using a Turner 20/20 Luminometer. Firefly reporter activities were normalised using Renilla luciferase values. Normalised values for mutant Tax proteins were calculated as a percentage of wildtype Mo values which was set at 100%.

**Table 3:** Transactivation phenotypes of Tax 2A Mo and Tax 1 mutants

	Mutation	LTR	NFkB
Mo	None	100%	100%
	G21D	14%	140%
	L87I	< 5%	125%
	P92L	13%	120%
	Y144C	< 5%	< 5%
	Y144R	< 5%	< 5%
	T204A	300%	168%
	W248R	10%	< 5%
	L308V	75%	80%
2B	None	246%	195%
Tax 1	None	100%	100%
	Y144C	55%	36%
	W248R	< 5%	< 5%

Jurkat cells were co-transfected with the indicated Tax plasmids together with 1 ug of the LTR or NFkB luciferase reporters and 50 ng of the control Renilla plasmid pRL TK. After 24 hrs the cells were lysed and reporter activities were measured using a Turner 20/20 Luminometer and firefly reporter activities were normalised using Renilla luciferase values. Normalised values for wildtype Tax 2B and mutant Tax proteins were calculated as a percentage of the corresponding wildtype Mo or Tax 1 values which was set at 100%.

### Transactivation phenotypes of Tax 2B mutants

Given the high degree of homology between Tax2A and Tax 2B we sought to compare functional domains in both proteins by introducing the mutations found in Gar and Lor into Tax 2B (Table 4). In a similar manner to its effect on Mo and Tax 1, W248R abolished the ability of 2B to activate either the LTR or NFkB promoters and similar to its effects on Tax 1 Y144C appeared to only reduce the activity of Tax 2B. However the introduction of an arginine instead of a cysteine at this position (Y144R) into Tax 2B abolished its activity indicating that this position is important for function but may depend on the amino acid present. Mutations at positions G21D, L87I, P92L and L308V appeared to have similar effects on Tax 2B activity as they had on the activity of Mo in as much as they substantially reduced LTR activation while not affecting the activation of NFkB. As was previously noted wildtype Mo was found to activate the CREB and NFkB pathways less efficiently than Tax 2B (Table 3). This difference was abolished by the introduction of the mutation T204A into Mo. An alanine occurs naturally at this position in Tax 2B, the mutation of which to a threonine (A204T) results in similar transactivation activities as Mo (Table 4). This indicates that this residue is responsible for the differences found in the activities of both proteins. All Tax 2B mutants, including 2B A204T (data not shown), were expressed at levels similar to wildtype Tax 2B except for W248R which appeared to be poorly expressed in a manner similar to Mo W248R.

**Table 4:** Transactivation phenotypes of Tax 2B mutants

	Mutation	LTR	NFkB
2B	None	100%	100%
	G21D	26%	138%
	L87I	< 10 %	90%
	P92L	24%	86%
	Y144C	58%	65%
	Y144R	< 5%	< 5%
	A204T	41%	54%
	W248R	< 5%	< 5%
	L308V	66%	88%
Mo	None	41%	73%

Jurkat cells were co-transfected with either wildtype Tax 2B or Tax 2B mutants together with 1 ug of the LTR or NFkB luciferase reporters and 50 ng of the control Renilla plasmid pRL TK. After 24 hrs the cells were lysed and reporter activities were measured using a Turner 20/20 Luminometer and firefly reporter activities were normalised using Renilla luciferase values. Normalised values for wildtype Mo and mutant Tax proteins were calculated as a percentage of wildtype 2B values which was set at 100%.

## Discussion

Even though Tax 1 and Tax 2 share approximately 70% homology, previous studies comparing the activities of Tax 1 and Tax 2 proteins have shown that functional differences exist between the two proteins and suggest that this could account at least in part for differences in the pathogenic properties of HTLV-1 and HTLV-2 [23]. Specifically Tax 2A was reported to be unable to induce micronuclei formation or to activate the ICAM-1 promoter in T cells compared to Tax 1 [24,25]. Furthermore while all Tax proteins inhibit p53 activity, Tax 2A was found to do so less efficiently than either Tax I or Tax 2B [26]. In transformation studies, Tax 2A was found to transform primary human T cells with the same efficiency as Tax 1 and while Tax 2A and Tax 2B could transform Rat-1 cells they did so less efficiently than Tax 1 [27]. Other studies showed that in contrast to Tax 2, Tax 1 suppressed hematopoiesis in transduced CD34+ progenitor cells and suggested that this may be attributed to its ability to upregulate the cyclin-dependent kinase inhibitor p21^cip/waf1 ^promoter more efficiently than Tax 2 [28,29]. In addition Jurkat cells that constitutively express Tax 1 were shown to inhibit the kinetics of cellular replication to a higher degree compared to Tax 2 [30]. In the present study we investigated the ability of two Tax 2A proteins Lor and Gar to transactivate the LTR and NFkB promoters in relation to expression levels, sequence variation and sub cellular location compared to Tax 2A Mo and Tax 2B. Lor and Gar failed to activate either promoter compared to Mo or 2B eventhough the expression levels of all Tax 2 proteins were similar. Compared to Mo, we identified six amino acid changes in Lor spanning the entire protein and one mutation in Gar located in the centre of the protein. Lor was derived from a HTLV-2A infected BJAB cell line which was positive for p24 production by FACS analysis (data not shown) indicating that the mutations present were not affecting the function of Rex. It was not possible to determine if the amino acid changes in Lor arose during culture or if they were present in the original virus. A previous study found that compared to Mo the prevalence of amino acid changes in some functional Tax 2A proteins was low (1–2%) [31] which is similar to that found in the non-functional Lor protein. The Tax cDNAs in that study were derived from non-cultured PBMCs obtained from infected individuals thus eliminating the possibility that the mutations arose as a result of cell culture. Examination of those Tax 2A sequences revealed that they included only one of the mutations described in the present study, at position T204A which appears to be present in most Tax 2A sequences.

In the present study most of the individual mutations appeared only to affect the ability of Mo to activate the LTR and had little affect on NFkB activation. The amino terminal mutations are located in previously described functional domains in Tax 1 and Tax 2 proteins including a nuclear localization signal, zinc finger domain and more recently a domain in Tax 2 between amino acids 90–100 shown to be involved in the cytoplasmic location of Tax 2 proteins [13,14,16,22]. However analysis of the subcellular location of mutant proteins using immunofluorescence did not reveal any discernable alterations in their distribution compared to wildtype Mo. We found that all Tax 2 proteins were predominantly located in the cytoplasm and also to a lesser extent in the nucleus. These results agree with a recent study where they also found that in contrast to Tax 1, Tax 2 proteins are predominantly found in the cytoplasm [22]. Two mutations in the central region of Tax 2A at positions 144 and 248 appeared to abolish both LTR and NFkB activation indicating that these mutations may disrupt an essential functional or structural domain involved in the activation of both pathways by Mo. The mutation at position W248R resulted in defective LTR and NFkB activation both in the presence of other mutations in Lor and when it is introduced singly into Mo. The replacement of this mutation with the corresponding wildtype residue in the Lor mutant L6 restored a wildtype NFkB phenotype but resulted in defective LTR activation. The overall phenotype of L6 was probably due to the combined effects of the other Lor mutations present in the L6 protein which individually were found to substantially reduce LTR activation by Mo without affecting activation of the NFkB pathway. A mutation in close proximity to 248, at position 258, was described in previous studies to abolish Tax 2A activity while Tax 1 containing this mutation failed to transactivate NFkB but retained the capacity to transactivate the HTLV-1 LTR [13,19]. In the present study insertion of the mutation at position 248 into both Tax 1 and 2B also abolished their activity indicating that this mutation may disrupt a shared functional or structural domain required for activation of both pathways by all three Tax proteins. As opposed to its effects on Mo, the mutation at position Y144C only reduced the ability of Tax 2B and Tax 1 to activate the LTR and NFkB promoters indicating that this domain is not as critical in Tax 1 and 2B for activity as it is in Mo. However the insertion of the amino acid arginine instead of the hydrophobic amino acid cysteine at this position abolished Tax 2B activity. It is not clear why the expression of some Tax mutants, such as Mo W248R and Tax 2B W248R, was substantially reduced compared to the corresponding wildtype proteins. This is in contrast to the expression levels of Lor and Lor mutant proteins L1–L4, which were not affected by the presence of W248R. Wildtype Mo was repeatedly found to activate both the LTR and NFkB promoters less efficiently than Tax 2B. However this difference was abolished by introducing one mutation at position T204A into Mo which resulted in similar or slightly higher levels of activity to those obtained with wildtype Tax 2B indicating that depending on sequence of both proteins, Mo and Tax 2B display equivalent levels of activity. These results differ from a previous study carried out in our laboratory which found that compared to Tax 1 and Tax 2B some Tax 2A proteins including Mo were unable to activate the CREB pathway in Jurkat or 293T cells [12]. We speculate that these differences may be related to the poor expression of Tax 2A proteins reported in that study and possibly to differences in experimental conditions.

## Conclusion

In conclusion, the present study shows that compared to Mo, certain Tax 2A proteins are non-functional and that loss of activity is clearly associated with the accumulation of amino acid changes and not with levels of expression or alterations in sub-cellular localisation. Failure of Tax 2A mutants to activate either the CREB or NFkB pathways or both, was previously reported to be related to an inability to transform T cells [32]. This, together with our findings, suggests that the prevalence of mutations in Tax 2A proteins which inactivate both pathways may influence the pathogenic properties of certain HTLV-2A viruses.

## Materials and methods

### Cell lines and plasmids

293T and Cos 7 cells were maintained in Dulbecco's minimal essential medium (DMEM) supplemented with 10% foetal bovine serum and Penicillin/Streptomycin and Gentamicin. Jurkat E6-1 T cells were maintained in RPMI-1640 medium supplemented with 10% foetal bovine serum and Penicillin/Streptomycin and Gentamicin. To allow the simultaneous detection of all Tax proteins using a single antibody, Tax coding sequences were amplified by PCR using reverse primers that contained an additional sequence for six histidine (HIS) residues before the stop codons. For cloning purposes all primers contained 5' and 3' EcoRI restriction enzyme sites. Tax 2A Lor was amplified by PCR from genomic DNA extracted from an HTLV-2A infected BJAB cell line. Gar was amplified from genomic DNA extracted from cultured PBMCs from a HTLV-2/HIV-1 infected individual and Mo was amplified from a plasmid construct supplied by P.L. Green. Tax 1 and Tax 2B coding sequences were amplified from the corresponding pFLAG constructs as described previously [12]. Purified PCR products were cloned into the mammalian expression plasmid pCAGGS using EcoRI. The nucleotide sequence of all constructs was determined using the BigDye Terminator sequencing kit (Applied Biosystems). The HTLV-1 LTR luciferase plasmid were described previously [12] and NFkB activation was determined using pNF-kB-Luc (Stratagene).

### Transient transfections and luciferase assays

Plasmid DNA was introduced into cells using Fugene tranfection reagent (Roche Diagnostics) according to the manufacturer's instructions. For functional assays, 1 × 10^5 ^Jurkat cells were seeded in 60 mm dishes and co-transfected with either 1 ug of HTLV-1 LTR, or NFkB firefly luciferase reporters together with 250 ng of the indicated Tax expression plasmids and 50 ng of Renilla luciferase reporter pRL-TK. Reporter activities were measured using the Dual Luciferase reporter assay system (Promega) 24 hrs after transfection as described previously. Briefly, cells were lysed in 1× passive lysis buffer and firefly and Renilla luciferase activities were measured using a Turner 20/20 Luminometer. Reporter activities were normalized using Renilla luciferase values. To determine and compare Tax expression levels in cells transfected with wildtype or mutant plasmids, 293T cells were seeded on 60 mm dishes and co-transfected the next day with 250 ng of the indicated plasmids. Cells were lysed after 24 hrs using 1× passive lysis buffer. Lysates was analysed by western blotting and Tax proteins were detected using an anti-HIS antibody (Invitrogen). Blots were also probed with anti-Tubulin (Calbiochem) as a loading control.

### Site directed mutagenesis

Point mutations in Tax 1, Tax 2A and Tax 2B constructs were generated using the QuickChange Site Directed Mutagenesis kit (Stratagene) according to the manufacturers instructions. The presence of mutations was confirmed by sequencing using the BigDye Terminator sequencing kit (Applied Biosystems).

### Indirect immunofluorescence

Cos 7 cells were seeded on two well chamber slides twenty four hrs before transfection with 150 ng of the indicated Tax expression plasmids. Twenty four hours after transfection cells were washed with PBS, fixed with 4% paraformaldehyde for 20 min at room temperature and permeabilized in 0.2% Tween 20/PBS. Non specific binding was blocked using 5% rabbit serum or swine serum for 1 h at room temperature and incubated with the anti-HIS antibody (Invitrogen; 1:400) for 2 h at room temperature. After washing in PBS, cells were incubated with rabbit anti- mouse FITC for 1 h at room temperature. Following a washing step the nuclei in cells were stained using DAPI (Sigma 1 ug/ml) and slides were mounted in Vectashield.

## Competing interests

The author(s) declare that they have no competing interests.

## Authors' contributions

NS carried out the site directed mutagenesis and performed the functional assays. LL made the Tax expression plasmid Lor. ML provided the Tax 1, Tax 2B, Gar plasmids which were used as templates to amplify Tax genes that were used to construct the expression plasmids in the present study. KW provided technical assistance and VG provided useful suggestions. All authors read and approved the final manuscript.

## References

[B1] Poiesz BJ, Ruscetti FW, Gazdar AF, Bunn PA, Minna JD, Gallo RC (1980). Detection and isolation of type C retrovirus particles from fresh and cultured lymphocytes of a patient with cutaneous T-cell lymphoma. Proc Natl Acad Sci USA.

[B2] Yoshida M, Miyoshi I, Hinuma Y (1982). Isolation and characterization of retrovirus from cell lines of human adult T-cell leukemia and its implication in the disease. Proc Natl Acad Sci USA.

[B3] Gessain A, Barin F, Vernant JC, Gout O, Maurs L, Calender A, de The G (1985). Antibodies to human T-lymphotropic virus type-I in patients with tropical spastic paraparesis. Lancet.

[B4] Osame M, Usuku K, Izumo S, Ijichi N, Amitani H, Igata A, Matsumoto M, Tara M (1986). HTLV-1 associated myelopathy, a new clinical entity. Lancet.

[B5] Roucoux DF, Murphy EL (2004). The epidemiology and disease outcomes of human T-lymphotropic virus type II. AIDS Rev.

[B6] Araujo A, Hall WW (2004). Human T-lymphotropic virus type II and neurological disease. Ann Neurol.

[B7] Hall WW, Ishak R, Zhu SW, Novoa P, Eiraku N, Takahashi H, Ferreira Mda C, Azevedo V, Ishak MO, Ferreira Oda C, Monken C, Kurata T (1996). Human T lymphotropic virus type II (HTLV-2): epidemiology, molecular properties, and clinical features of infection. J Acquir Immune Defic Syndr Hum Retrovirol.

[B8] Azran I, Schavinsky-Khrapunsky Y, Aboud M (2004). Role of Tax protein in human T-cell leukemia virus type-I leukemogenicity. Retrovirology.

[B9] Eiraku N, Novoa P, da Costa Ferreira M, Monken C, Ishak R, da Costa Ferreira O, Zhu SW, Lorenco R, Ishak M, Azvedo V, Guerreiro J, de Oliveira MP, Loureiro P, Hammerschlak N, Ijichi S, Hall WM (1996). Identification and characterization of a new and distinct molecular subtype of human T-cell lymphotropic virus type 2. J Virol.

[B10] Lewis MJ, Novoa P, Ishak R, Ishak M, Salemi M, Vandamme AM, Kaplan MH, Hall WW (2000). Isolation, cloning, and complete nucleotide sequence of a phenotypically distinct Brazilian isolate of human T-lymphotropic virus type II (HTLV-2). Virology.

[B11] Vandamme AM, Salemi M, Van Brussel M, Liu HF, Van Laethem K, Van Ranst M, Michels L, Desmyter J, Goubau P (1998). African origin of human T-lymphotropic virus type 2 (HTLV-2) supported by a potential new HTLV-2d subtype in Congolese Bambuti Efe Pygmies. J Virol.

[B12] Lewis MJ, Sheehy N, Salemi M, VanDamme AM, Hall WW (2002). Comparison of CREB- and NF-kappaB-mediated transactivation by human T lymphotropic virus type II (HTLV-2) and type I (HTLV-1) tax proteins. Virology.

[B13] Smith MR, Greene WC (1990). Identification of HTLV-1 tax trans-activator mutants exhibiting novel transcriptional phenotypes. Genes Dev.

[B14] Semmes OJ, Jeang KT (1992). Mutational analysis of human T-cell leukemia virus type I Tax: regions necessary for function determined with 47 mutant proteins. J Virol.

[B15] Smith MR, Greene WC (1992). Characterization of a novel nuclear localization signal in the HTLV-1 tax transactivator protein. Virology.

[B16] Turci M, Romanelli MG, Lorenzi P, Righi P, Bertazzoni U Localization of human T-cell lymphotropic virus type II Taxprotein is dependent upon a nuclear localization determinant in the N-terminal region. Gene.

[B17] Alefantis T, Barmak K, Harhaj EW, Grant C, Wigdahl B (2003). Characterization of a nuclear export signal within the human T cell leukemia virus type I transactivator protein Tax. J Biol Chem.

[B18] Chevalier SA, Meertens L, Calattini S, Gessain A, Kiemer L, Mahieux R (2005). Presence of a functional but dispensable nuclear export signal in the HTLV-2 Tax protein. Retrovirology.

[B19] Ross TM, Minella AC, Fang ZY, Pettiford SM, Green PL (1997). Mutational analysis of human T-cell leukemia virus type 2 Tax. J Virol.

[B20] Burton M, Upadhyaya CD, Maier B, Hope TJ, Semmes OJ (2000). Human T-cell leukemia virus type 1 Tax shuttles between functionally discrete subcellular targets. J Virol.

[B21] Szymocha R, Akaoka H, Brisson C, Beurton-Marduel P, Chalon A, Bernard A, Didier-Bazes M, Belin MF, Giraudon P (2000). Astrocytic alterations induced by HTLV type 1-infected T lymphocytes: a role for Tax-1 and tumor necrosis factor alpha. AIDS Res Hum Retroviruses.

[B22] Meertens L, Chevalier S, Weil R, Gessain A, Mahieux R (2004). A 10-amino acid domain within human T-cell leukemia virus type 1 and type 2 tax protein sequences is responsible for their divergent subcellular distribution. J Biol Chem.

[B23] Feuer G, Green PL (2005). Comparative biology of human T-cell lymphotropic virus type 1 (HTLV-1) and HTLV-2. Oncogene.

[B24] Semmes OJ, Majone F, Cantemir C, Turchetto L, Hjelle B, Jeang KT (1996). HTLV-1 and HTLV-2 Tax: differences in induction of micronuclei in cells and transcriptional activation of viral LTRs. Virology.

[B25] Tanaka Y, Hayashi M, Takagi S, Yoshie O (1996). Differential transactivation of the intercellular adhesion molecule 1 gene promoter by Tax1 and Tax2 of human T-cell leukemia viruses. J Virol.

[B26] Mahieux R, Pise-Masison CA, Lambert PF, Nicot C, De Marchis L, Gessain A, Green P, Hall W, Brady JN (2000). Differences in the ability of human T-cell lymphotropic virus type 1 (HTLV-1) and HTLV-2 tax to inhibit p53 function. J Virol.

[B27] Endo K, Hirata A, Iwai K, Sakurai M, Fukushi M, Oie M, Higuchi M, Hall WW, Gejyo F, Fujii M (2002). Human T-cell leukemia virus type 2 (HTLV-2) Tax protein transforms a rat fibroblast cell line but less efficiently than HTLV-1 Tax. J Virol.

[B28] Tripp A, Liu Y, Sieburg M, Montalbano J, Wrzesinski S, Feuer G (2003). Human T-cell leukemia virus type 1 tax oncoprotein suppression of multilineage hematopoiesis of CD34+ cells in vitro. J Virol.

[B29] Tripp A, Banerjee P, Sieburg M, Planelles V, Li F, Feuer G (2005). Induction of cell cycle arrest by human T-cell lymphotropic virus type 1 Tax in hematopoietic progenitor (CD34+) cells: modulation of p21cip1/waf1 and p27kip1 expression. J Virol.

[B30] Sieburg M, Tripp A, Ma JW, Feuer G (2004). Human T-cell leukemia virus type 1 (HTLV-1) and HTLV-2 tax oncoproteins modulate cell cycle progression and apoptosis. J Virol.

[B31] Hjelle B, Chaney R (1992). Sequence variation of functional HTLV-II tax alleles among isolates from an endemic population: lack of evidence for oncogenic determinant in tax. J Med Virol.

[B32] Ross TM, Narayan M, Fang ZY, Minella AC, Green PL (2000). Human T-cell leukemia virus type 2 tax mutants that selectively abrogate NFkappaB or CREB/ATF activation fail to transform primary human T cells. J Virol.

